# Atypical CHARGE associated with a novel frameshift mutation of *CHD7* in a Chinese neonatal patient

**DOI:** 10.1186/s12887-018-1181-0

**Published:** 2018-06-26

**Authors:** Yan-ping Xu, Li-ping Shi, Jiajun Zhu

**Affiliations:** 1 NICU, Children’s Hospital, Zhejiang University School of Medicine, Hangzhou, 310053 China; 2 Department of Neonatology, Women’s Hospital, Zhejiang University School of Medicine, Hangzhou, 310006 China

**Keywords:** CHARGE syndrome, Choanal atresia, CHD7

## Abstract

**Background:**

CHARGE syndrome is an autosomal dominant malformation disorder caused by heterozygous loss of function mutations in the chromatin remodeler *CHD7*, which has been estimated to occur in 1:10,000 births worldwide. It is a genetic disorder closely resembles other pattern of anomalies. Genetic testing should be pointed out as a useful method for clinical diagnosis.

**Case presentation:**

A female infant was the second child born to a 33-year-old, gravida 3, para 2 mother. The infant was born at 37 + 4 weeks of gestation with a birth weight of 2440 g (− 1.1 S.D.). Clinical examination showed atypical CHARGE syndrome, with choanal atresia, a heart defect, and sensorineural deafness. Genomic DNA was extracted from peripheral venous blood sample using molecular biological technique. We used the Illumina TruSigt One sequencing panel on the MiSeq next- generation sequencing (NGS) platform for mutation screening and found a novel frameshift mutation in chromodomain helicase DNA binding protein 7 (*CHD7*; c.4656dupT)**.** This mutation results in a new reading frame ending in p.(Ile1553fs). At the first month of age, the patient had a posterior nostril plasty operation by nasal endoscope. At the second month of age, she had patent ductus arteriosus ligation surgery. At the 4th month of age, she was discharged from the hospital.

**Conclusions:**

Our findings further reveal that patients should not be rejected for CHD7 mutational analysis even if they do not fulfill CHARGE syndrome Verloes criteria.

## Background

CHARGE syndrome is a complex genetic disorder, which has been estimated to occur in 1:10,000 births worldwide and shows various clinical manifestations, causing multiple birth defects and sensory deficits [1]. The pattern of anomalies now associated with CHARGE syndrome was first recognized in 1979 by Hittner et al. [2] and Hall [3]. The major clinical features of CHARGE syndrome (OMIM 214800) are ocular **C**oloboma, congenital **H**eart defects, choanal **A**tresia, **R**etardation of growth, **G**enital hypoplasia, and **E**ar abnormalities. CHARGE syndrome closely resembles other pattern of anomalies, so genetic testing should be emphasized as a useful method in clinical diagnosis. Of all the clinically diagnosed CHARGE patients, 67 to 90% have been shown to have pathogenic mutations in the gene encoding chromodomain helicase DNA binding protein 7 (*CHD7,* OMIM *608892), which is located on chromosome 8q12.1. Detailed information on the variant in *CHD7* gene reported so far, approximately 70% are nonsense or frameshift, 10% are splice site, 15% are missense, and 5% are whole-gene or chromosomal deletion, exonic deletion and chromosomal rearrangement [4]. Only a few studies have been reported that mutation in *EFTUD2* (OMIM 603892) at chromosome 17q21 may also cause CHARGE like syndrome [5, 6]. Here, we presented a novel monoallelic frameshift mutation of *CHD7*, NM_017780.3 (c.4656dupT) in a Chinese patient with CHARGE syndrome.

## Case presentation

We describe the case of a female infant. She was the second child born to a 33-year-old, gravida 3, para 2 mother. The patient was born polyhydramnios by cesarean section at 37 + 4 weeks of gestation with a birth weight of 2440 g (− 1.1 S.D.), a length of 50 cm (+ 0.80 S.D.) and an occipitofrontal circumference of 36 cm (+ 2.0 S.D.). The 1- and 5-min Apgar scores were 8 and 8, respectively. Shortly after birth, she required nasal continuous positive airway pressure (nCPAP) and presented with dyspnea. During the following days, she developed dyspnea continually and needed oxygen to maintain 90–95% saturation. Parenteral nutrition was started on day 1 and breast milk was given 12 h after birth by oral tube. Her parents were nonconsanguineous and her mother had a healthy 13-year-old child. She denied any family history of neonatal disease. Prenatal examination was not found abnormal. Additionally, she denied that she had consumed alcohol, drugs, tobacco, or any other toxic substances during her pregnancy.

**Fig. 1:**
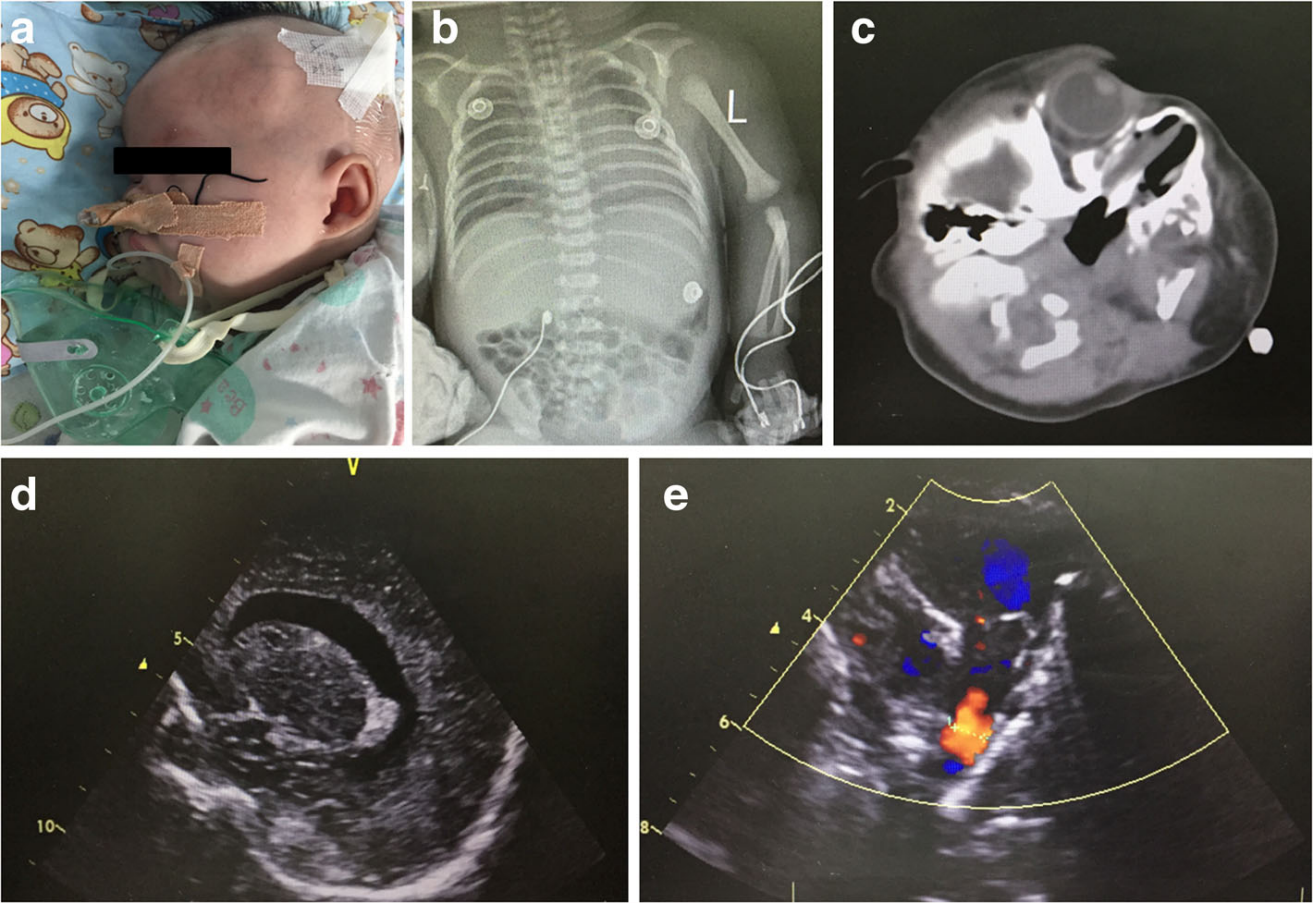
**a** Patient did posterior nostril plasty operation by nasal endoscope and put silicone tube in one month. **b** Chest radiograph at 3 days. **c** Computed tomography revealed bilateral choanal atresia and semi-circular canals as an insufficient inflatable structure. **d** Craniocerebral ultrasound showed bilateral lateral ventricle dilatation. **e** Echocardiography showed atrioventricular septal defect (6.8 mm + 2.2 mm) and patent ductus arteriosus (3.6 mm)

On admission to our unit, the patient was 3 days old and weighed 2400 g. Clinical examination showed choanal atresia, bilateral low-set ears, triple restriction and systolic murmur, but coloboma was not observed. Her motor development was almost normal. The patient presents feeding difficulties by nasogastric tube. Her white blood cell count was 12.07 × 109/L (neutrophils, 0.50; lymphocytes, 0.24), and her platelet count was 160.00 × 109/L and CRP < 1 mg/L. The alanine aminotransferase level was 14 U/L, aspartate aminotransferase level was 43 U/L, and gamma-glutamyltransferase level was 68 U/L. On the seventh day of age, her thyroid functional parameters were TSH 5 mIU/L, T3 1.83 nmol/L and T4 123.94 nmol/L, and at the first month of age, thyroid functional parameters were TSH > 100 mIU/L, T3 1.57 nmol/L and T4 35.93 nmol/L. Thus, oral Euthyrox (Levothyroxine sodium tablets) was administered. Newborn screening for metabolic disorders and severe combined immunodeficiency was normal. A chest radiograph showed haziness in both lung fields suggestive of wet lung (Fig. 1b). Two-dimensional and color-Doppler assessment was revealed an atrioventricular septal defect (6.8 mm + 2.2 mm), patent ductus arteriosus (3.6 mm) and pulmonary hypertension (Fig. 1e). A craniocerebral ultrasound showed bilateral lateral ventricle dilatation (Fig. 1d). The auditory brainstem response (ABR) test showed bilateral severe hearing impairment (ABR > 99 dBnHL). Following radiologic testing by computed tomography showed bilateral choanal atresia and insufficient inflatable structure of both semi-circular canals. (Fig. 1c). No clinical characteristics of CHARGE syndrome were detected in the patient’s parents. Chromosomal analysis indicated a 46 XX normal female karyotype.

The patient required mechanical ventilation with endotracheal intubation at 4 days of age. On 17 days of age, she was extubated to nCPAP with FiO2 < 25%. On 20 days of age, she needed an oxygen mask. Ampicillin was discontinued when the blood culture from birth was sterile at 72 h. At the first month of age, the patient had a posterior nostril plasty operation by nasal endoscope and had a silicone tube in one month for transition the postoperative (Fig. 1a). At the second month of age, she had patent ductus arteriosus ligation surgery. At the 4th month of age, she was discharged from the hospital. Clinical features summarized in Table 1.

**Table 1:** Clinical features and mutation of patients with mutation in the CHD7 gene

Sex	Age	Coloboma	Choanal atresia	Cleft lip and/or palate	SCC hypoplasia	Deafness	Feeding difficulties	Structural brain anomalies	Genital hypoplasia	Growth retardation	Heart defect	Kidney anomalies	IUGR	Classfication	Mutation
F	3 d	–	+	–	–	+	+	–	–	–	+	–	–	Atypical CHARGE	*CHD7* c.4656dupT

*SCC* semicircular canal

**Fig. 2:**
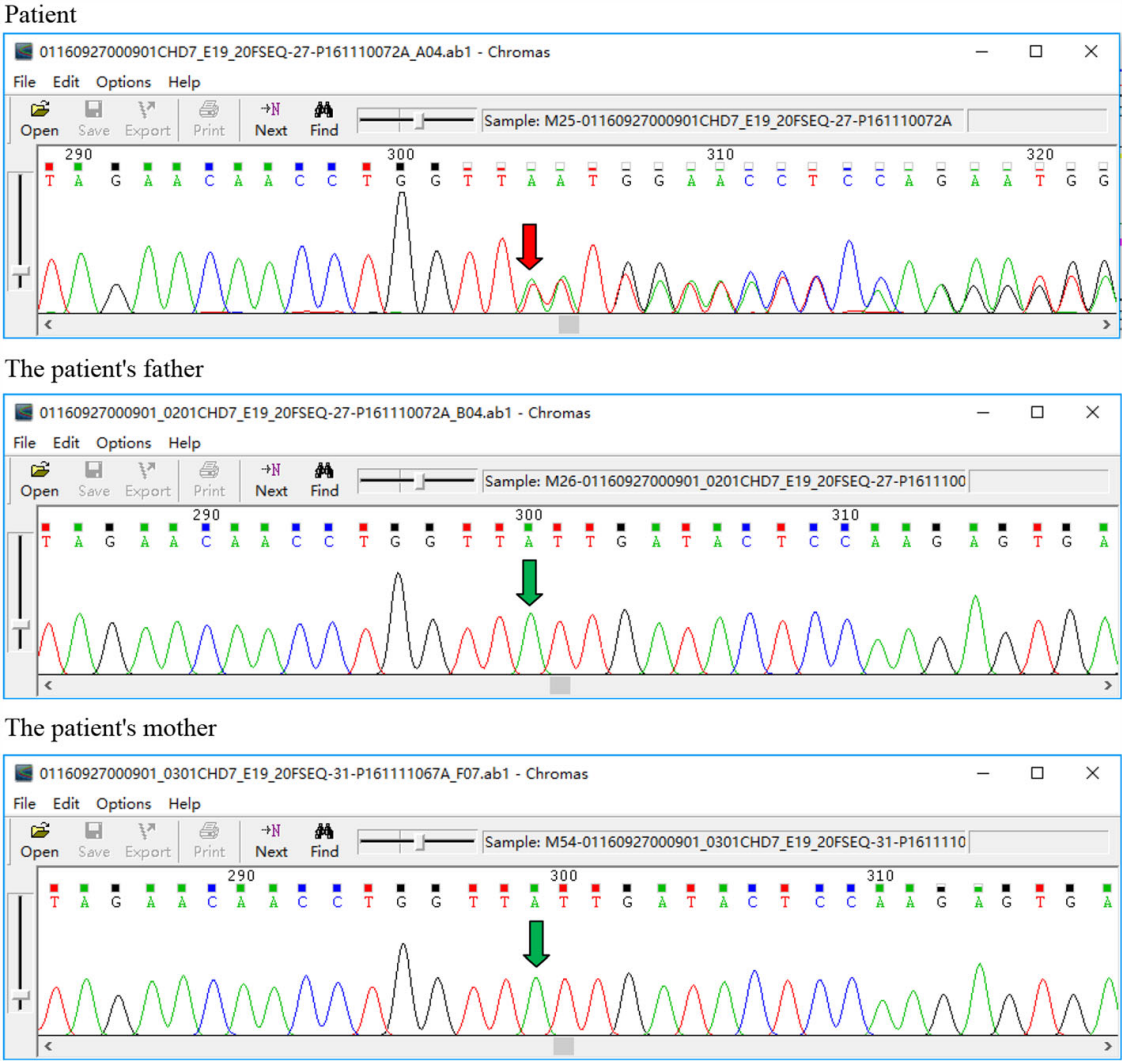
Results of monoallelic frameshift mutation in *CHD7*, NM_017780.3 (*CHD7* c.4656dupT), this mutation causes a frameshift starting from isoleucine, with the new reading frame ending p.(Ile1553fs)

Molecular analysis of the disease-associated genes *CHD7* and *EFTUD2* were performed using the Illumina TruSigt One sequencing panel (Illumina, San Diego, CA, USA) on the MiSeq NGS platform for mutation screening methods (Sinopath Diagnosis, Beijing, China) [7]. Genomic DNA was extracted from peripheral venous blood and informed consent was obtained from the parents. The study was approved by the ethics committee of Zhejiang University Children’s Hospital. To identify presumably pathogenic single-nucleotide variants, we used NextGene V2.3.4 (Softgenetics, State College, PA, USA) compared with the UCSC database. We excluded sequence variants with a minor allele frequency > 0.05, in the Human Genetic Variation Database (http://www.genome.med.kyoto-u.ac.jp/SnpDB/) and the NHLBI Grand Opportunity Exome Sequencing Project (ESP6500, http://evs.gs.washington.edu/EVS/). This analyses identified a monoallelic (thymine) insertion in *CHD7*, NM_017780.3 (*CHD7* c.4656dupT), which was confirmed by Sanger sequencing. This mutation leads to a reading frameshift mutation starting from isoleucine, with the new reading frame ending p.(Ile1553fs) (Fig. 2). As shown in Fig. 2, the mutation is located in exon 20, and this mutation was not present in the Human Gene Mutation Database (http://www.hgmd.org/) or ClinVar (http://www.ncbi.nlm.nih.gov/clinvar/),[8] suggesting that it is novel. This heterozygous frameshift mutation was not detected in the patient’s parents, suggesting that it is a de novo mutation.

## Discussion and conclusions

Applying the scoring scheme by Verloes, a patient can be assigned to atypical CHARGE syndrome, if they have one major criterion (choanal atreisia) and three minor criteria (heart malformation, deafness and external ear malformations) [9]. Low set ears are not a typical feature of CHARGE syndrome. Abnormal outer ears always like cup-shaped in the typical CHARGE syndrome. The aetiology remains unknown. In the second screening of thyroid function (at the age of 1 mo), the patient was diagnosed with hypothyroidism. It has been reported that four patients had hypothyroidism combined with CHARGE syndrome: two had central hypothyroidism with a low response to the thyrotropin-releasing hormone loading test, while the others had primary hypothyroidism and received thyroxine replacement. Some cases also had Growth Hormone Deficiency (GHD) and used growth hormone therapy [10]. This is a reason for the presentation of development delay and growth retardation. However, the concentration of growth hormone was not assessed in the neonatal period, and we could not determine the precise frequency of GHD in our patient. Typical features of CHARGE syndrome is well diagnosed, but the atypical part of the disease is difficult to identify. A person with subtle symptoms can pass their mutations on to offspring who is associated with a more severe phenotype. It is important to provide such patients with accurate prognostic information and genetic counseling. Additionally, compared to children or adults, features of CHARGE syndrome in neonates are atypical and less, so analysis of disease-associated genes including *CHD7* and *EFTUD2* should be done in infants, who do not completely meet the major clinical criteria. We identified a monoallelic c.4656dupT insertion of *CHD7,* leading to a novel frameshift mutation and an early stop codon, which resulted in a truncated CHD7 protein. *CHD7* genomic structure spans 188 kb and consists of 38 exons, the first of which is noncoding.*CHD7* expression remains ubiquitous in later stages of fetal development. Problems appear early in the first trimester and specifically occur between the third and ninth weeks postconception [12, 13]. At multiple stages of embryonic development indicate that CHD7 is localized to specific in both tissue and stage affected for CHARGE syndrome including the developing eye, ear and olfactory system [4]. Sangar sequencing of CHD7 gene was used to detect mutations (point mutation, small deletions and/or insertions in exons) in infants who were suspected of CHARGE syndrome. However, the method may miss some cases. The technique of multiplex ligation dependent probe amplification was used as supplement to detect small exonic deletions. Studies showed that deficit in exon 7 of CHD7 gene was related to CHARGE syndrome [11, 12]. Chai M found that *CHD7* is required for epigenetic activation of superenhancers and central nervous system-specific enhancers. Furthermore, they found that *CHD7*, through its interactions with superenhancer elements, acts as a regulatory hub in the orchestration of the spatiotemporal dynamics of transcription factors to regulate human neuroepithelial and central nervous system lineage identities [14]. Okuno H found that the expression of genes associated with cell migration was altered in CHARGE iPSC-NCCs compared to control iPSC-NCCs. Their results support the historical inference that CHARGE syndrome patients exhibit defects in neural crest migration [15]. Our case had a *CHD7* frameshift mutation at exon 20 leading to an early stop codonpredicting the loss of about 50% of the protein. Further studies are needed to delineate the roles of *CHD7* in enhancer-mediated transcriptional regulation in CHARGE syndrome in tissues of various development stages and their tissue expression sites. Genetic counseling was important for parents, even before we confirmed the diagnosis of CHARGE syndrome, because it could give them the information of the disease, realize the meaning of further genetic research, and provide the support to family. Most infants with CHARGE syndrome may develop abnormal, with motor and/or language problems, because of multiple sensory deficits. It is essential for family to early refer to rehabilitative therapist. Intelligence Quotients are various in the infants with CHARGE syndorme [16]. In conclusion, we report a case of atypical CHARGE syndrome, with the clinical features of choanal atresia, a heart defect, and sensorineural deafness, caused by a novel frameshift mutation in exon 20 of *CHD7*, with the new reading frame ending p. (Ile1553fs). Additional screening of atypical cases will be facilitated by molecular diagnosis. It should be emphasized that patients should not be rejected for *CHD7* analysis if they do not fulfill all the major criteria of CHARGE syndrome Verloes criteria.

## Abbreviations

CHD7: chromodomain helicase DNA binding protein 7
nCPAP: nasal continuous positive airway pressure
